# Soil type recognition as improved by genetic algorithm-based variable selection using near infrared spectroscopy and partial least squares discriminant analysis

**DOI:** 10.1038/srep10930

**Published:** 2015-06-18

**Authors:** Hongtu Xie, Jinsong Zhao, Qiubing Wang, Yueyu Sui, Jingkuan Wang, Xueming Yang, Xudong Zhang, Chao Liang

**Affiliations:** 1 State Key Laboratory of Forest and Soil Ecology, Institute of Applied Ecology, Chinese Academy of Sciences, Shenyang 110164, China; 2 Key Laboratory of Pollution Ecology and Environmental Engineering, Institute of Applied Ecology, Chinese Academy of Sciences, Shenyang 110164, China; 3 College of Resources and Environment, Huazhong Agricultural University, Wuhan 430070, China; 4 College of Land & Environment, Shenyang Agricultural University, Shenyang 110866, China; 5 Key Laboratory of Mollisols Agroecology, Northeast Institute of Geography and Agroecology, Chinese Academy of Sciences, Haerbin 150081, China; 6 Greenhouse and Processing Crops Research Centre, Agriculture & Agri-Food Canada, Harrow, Ontario N0R 1G0, Canada; 7 National Field Research Station of Shenyang Agroecosystems, Shenyang 110016, China; 8 Institute for Genomic Biology, University of Illinois at Urbana-Champaign, Urbana, IL 61801, USA

## Abstract

Soil types have traditionally been determined by soil physical and chemical properties, diagnostic horizons and pedogenic processes based on a given classification system. This is a laborious and time consuming process. Near infrared (NIR) spectroscopy can comprehensively characterize soil properties, and may provide a viable alternative method for soil type recognition. Here, we presented a partial least squares discriminant analysis (PLSDA) method based on the NIR spectra for the accurate recognition of the types of 230 soil samples collected from farmland topsoils (0–10 cm), representing 5 different soil classes (Albic Luvisols, Haplic Luvisols, Chernozems, Eutric Cambisols and Phaeozems) in northeast China. We found that the PLSDA had an internal validation accuracy of 89% and external validation accuracy of 83% on average, while variable selection with the genetic algorithm (GA and GA-PLSDA) improved this to 92% and 93%. Our results indicate that the GA variable selection technique can significantly improve the accuracy rate of soil type recognition using NIR spectroscopy, suggesting that the proposed methodology is a promising alternative for recognizing soil types using NIR spectroscopy.

Soil is a heterogeneous mixture of minerals and organic matter created by long-term pedogenic processes, which result in a variety of distinct types with differing properties and qualities. A soil’s type determines key physical and chemical properties and provides vital context for agricultural and ecological processes involving that soil. Soil type has traditionally been determined through a time-consuming combination of field investigations and laboratory analyses, both of which require specialized well-trained expertise. One emerging alternative to these approaches is the use of infrared spectroscopy. Infrared spectroscopy is a technique that uses the infrared spectrum of adsorption, emission, photoconductivity of a material; this technique has been successfully applied in various fields, including investigation of agricultural products, foodstuffs and pharmaceutical products^1^. In soil science, infrared spectroscopy is an established technique for analyzing soil samples in a fast, cost-effective, non-destructive manner that does not require hazardous chemicals^2^,^3^,^4^. Features within the infrared spectrum can be empirically associated with various soil properties, such as soil organic matter, texture, clay mineralogy, nutrient availability, fertility, structure and microbial activity^2^,^5^,^6^,^7^,^8^,^9^.

While infrared spectroscopy is not a new technique, its utility has been greatly improved by recent advances in mathematical and statistical methods for extracting information from multivariate and spectral data^10^. Pattern recognition methods were developed to explore similarity among groups of multivariate data^11^ and have been employed to identify soil samples using mid-infrared photoacoustic spectroscopy (MIR-PAS)^4^ and near-infrared spectroscopy (NIR)^3^. Chemometrics methods used in identification and classification of soils include principal component analysis (PCA)^4^,^12^,^13^, partial least squares (PLS) and artificial neural networks (ANN)^14^, cluster analysis^15^, linear discriminant analysis (LDA)^16^, soft independent modeling of class analogy (SIMCA)^16^ and partial least squares discriminant analysis (PLSDA)^17^. Among these methods, PLSDA is particularly effective at classification tasks^18^,^19^ and has the capacity to deal with data multicollinearity^20^.

Analytical methods for spectral data must frequently contend with redundant or uninformative features. Variable selection attempts to identify the most informative subset of a large number of variables with the aim of minimizing errors and excluding unreliable or noisy data^21^,^22^. In addition, variable selection reduces model complexity, simplifying interpretation^22^,^23^. Well-performed variable selection can improve model quality during calibration and enhance its resulting predictive performance^21^,^24^. Among the numerous variable selection methods available for chemometric analysis, genetic algorithms (GA) have demonstrated superior efficiency in systems as diverse as biofuel composition analysis^25^, olive oil classification^26^, and detection of insect infestation^27^. GAs mimic natural biological evolution to stochastically sample “populations” of variables drawn from a larger variable pool, with the aim of selecting a combination of variables that performs best under some specified criterion^28^,^29^. GAs can be used to select subsets of features from spectral information, potentially enhancing the performance of subsequent classification methods^30^. PLSDA models may be built from variables selected by GA (GA-PLSDA). Despite the potential benefits of this approach, we are unaware of any previous studies that have applied the GA-PLSDA method to soil classification.

In this study, we combine NIR spectroscopy data with PLSDA to categorize five soil types sampled from farmland in northeast China and explore the effect of GA variable selection on prediction accuracy. The objectives of this study were: 1) to illustrate a fast and accurate methodology for recognizing soils using NIR spectroscopy; and 2) to explore the effect of GA variable selection on PLSDA model calibration and performance.

## Results and discussion

**Figure 1:**
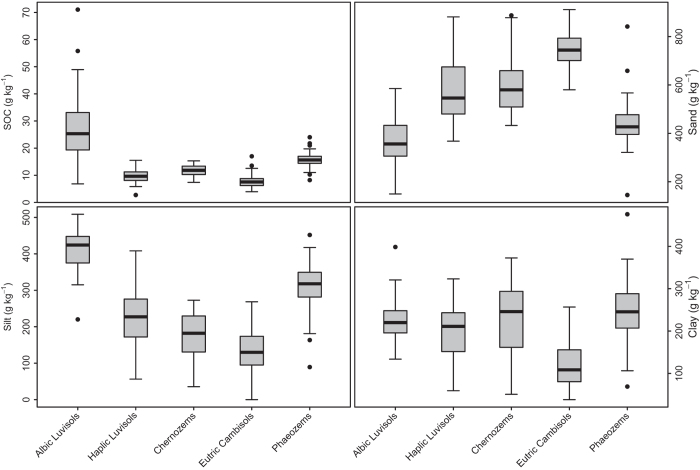
Ranges of soil organic carbon (SOC), sand, silt and clay contents found in five soil types in northeastern China.

**Figure 2:**
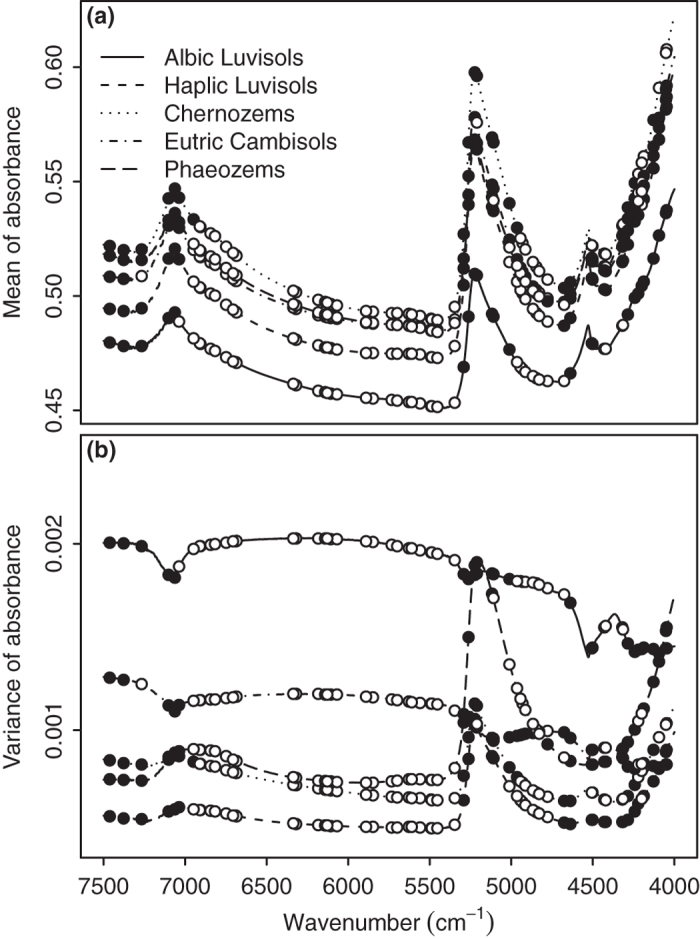
Mean and variance of NIR spectra for five different kinds of soil. The points indicate the variables in GA-PLSDA models. The solid ones represent the variables with VIP > 1.

The five soil types we studied differed in their texture and soil organic carbon (SOC) content (Fig. 1). The Albic Luvisols were characterized by high SOC and silt contents and a low sand content. Eutric Cambisols had the highest sand and lowest clay contents, while Phaeozems had the highest clay content and relatively high SOC and silt contents. There were general differences in NIR spectra among the soil types as well (Fig. 2a). Chernozems had the highest average absorbance across the spectrum, although they were similar to Phaeozems and Eutric Cambisols, while Albic Luvisols had the lowest average absorbance (Fig. 2a). Within-type variance in absorbance of specific wavenumbers also differed among soil types (Fig. 2b). The different absorbance strength among samples is the research basis of using PLSDA models for prediction.

**Figure 3:**
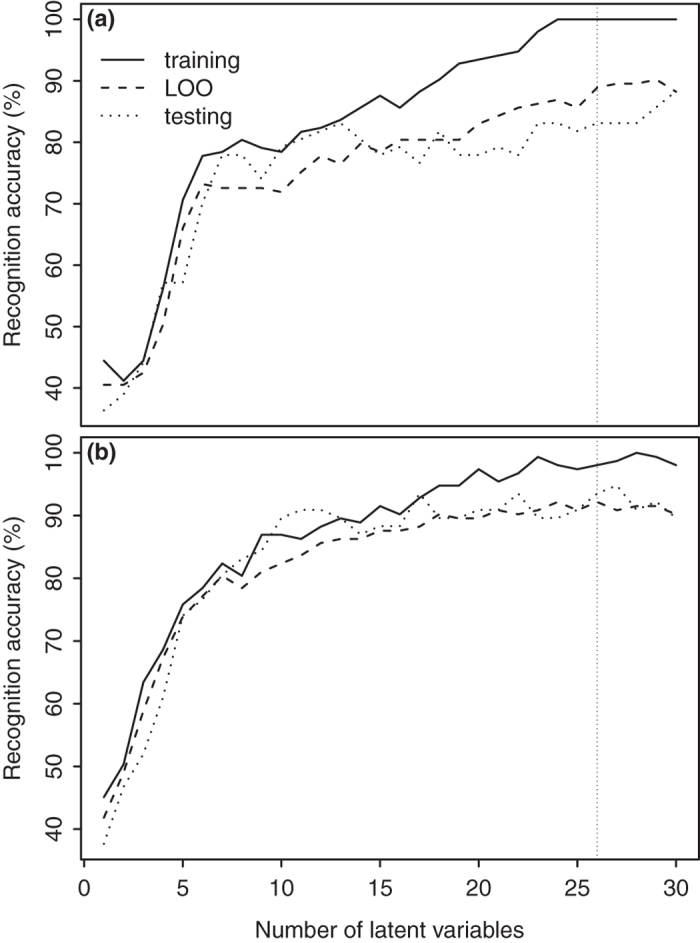
Classification accuracy for predicting soil type from near-infrared spectral data using (a) partial least squares discriminant analysis (PLSDA) and (b) PLSDA with a genetic algorithm (GA-PLSDA).

Using all 1816 NIR spectrum features, PLSDA achieved global internal validation (leave-one-out (LOO) cross validation) and external validation accuracies of 88.9% and 83.1%, respectively. LOO validation accuracy within soil types ranged from 63.2%-100.0% while external validation accuracy ranged from 62.5%-93.8% (Table 1). PLSDA was able to correctly classify all samples in the training set by using 26 latent variables, although external validation accuracy did not increase substantially above 13 variables (Fig. 3a).

The GA-PLSDA selected 66 out of 1816 available features (3.6%). This model attained 98.0%, 92.2%, and 93.5% training, LOO cross-validation, and external validation accuracies, respectively (Fig. 3b). Within soil types, accuracies ranged from 94.7%-100% for the training set, 79.0%-97.9% for LOO cross-validation, and 83.3%-100.0% for external validation (Table 2). Global internal and external validation accuracies were >90% with the GA-PLSDA with >20 latent variables (Fig. 3b).

In our study, the primary identifying NIR characteristic of various soil types were distributed in -OH region (7463-7037 cm^−1^; 5263-5222 cm^−1^; 4505-4503 cm^−1^), organics (5292 cm^−1^, 4320 cm^−1^ and 4316 cm^−1^), carbonate (4287 cm^−1^) and illite (4094 cm^−1^, 4050 cm^−1^ and 4046 cm^−1^). The -OH vibrations were predominant in each soil type, which is caused by vibrations of water bound in the interlayer lattices of clay minerals as hydrated cations and water adsorbed on particle surfaces^31^,^32^,^33^. The carbonate absorptions occur near 4287 cm^−1^ 33,34, mainly occurring from Chernozems and Eutric Cambisols. NIR spectra may reflect the differences in biogeographical origin^16^, organic composition^33^ and soil mineralogy. The relatively low rate of recognition accuracy for Haplic Luvisols (Table 1) was likely due to their geographic proximity with the Eutric Cambisls and Chemozems, or due to the fact that Haplic Luvisols shared pedogenesis (leached soils) with the Albic Luvisols^31^,^32^,^33^,^34^,^35^. Albic Luvisols sampled from piedmont were mostly discriminated from other soil types by mineral composition rather than SOC content. The minerals in Albic Luvisols soil are mainly mica-derived illite^35^. In general, relatively weak mineral weathering has occurred in northeast regions of China due to relatively dry and cold climatic conditions. As a result, minerals tend to be inherited from the parent material in those regions. Minerals of these five soils are typically clay mica (illite) (Albic Luvisols, Haplic Luvisols, Chernozems, Eutric Cambisols and Phaeozems), with small amounts of vermiculite (Haplic Luvisols and Eutric Cambisols soil), smectite (Eutric Cambisols, Chernozems), Kaolinite and unspecified minerals (Albic Luvisols soil)^35^,^36^.

We used variable importance in project (VIP) scores to identify the features that were most important for characterizing each soil type. We defined VIP scores ≥1 as indicative of particularly important features for the model^17^. Among the 66 features selected by GA, there were 28, 30, 29, 32 and 28 with VIP scores ≥1 (solid points in Fig. 2a and Fig. 2b) for Albic Luvisols, Haplic Luvisols, Chernozems, Eutric Cambisols and Phaeozems, respectively. Features with high VIP scores indicate spectral regions that contributed substantially to the model^37^. Some of these regions are associated with water, specific minerals, organic matter, or other identifiable aspects of the soil^7^. Our results showed that the high VIP features were distributed around 7065 cm^−1^, 5222 cm^−1^ and 4527 cm^−1^. These important wavenumbers reflect the overtones and combinations in -OH, organics and minerals. Only 12 features had high VIP scores for all 5 soil types, suggesting that these soils may differ in the chemical structures that characterize them, rather than the relative amounts of a common set of structure.

**Figure 4:**
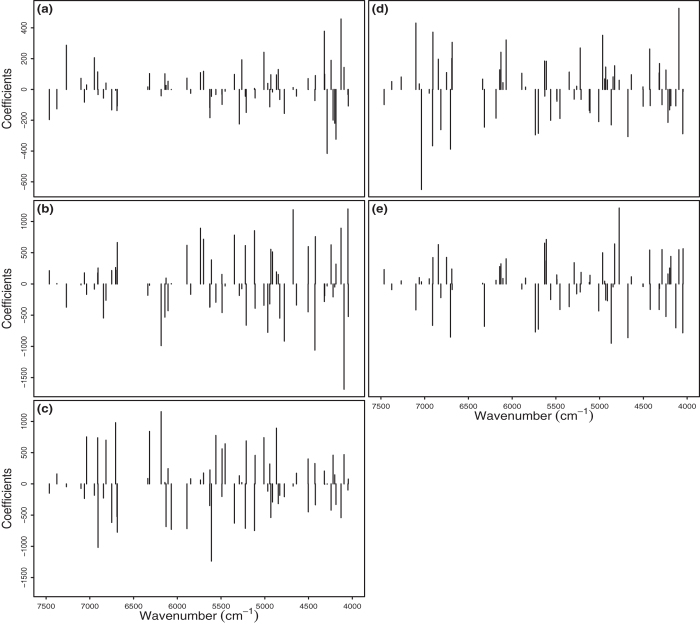
The coefficients of 66 variables selected by GA in the GA-PLSDA model. (**a**) Albic Luvisols; (**b**) Haplic Luvisols; (**c**) Chernozems; (**d**) Eutric Cambisols; (**e**) Phaeozems.

The importance of variables selected by GA can also be evaluated by the magnitude of their coefficients in the GA-PLDSA model. As with VIP scores, soils had distinct patterns of coefficient magnitudes (Fig. 4). For example, Albic Luvisols had a concentration of features with large coefficients in the range of 4500 - 4000 cm^−1^, while impactful features for Chernozems were evenly distributed across the spectrum. Variables whose coefficients have different signs in two soil types are particularly effective at discriminating between those soils.

**Figure 5:**
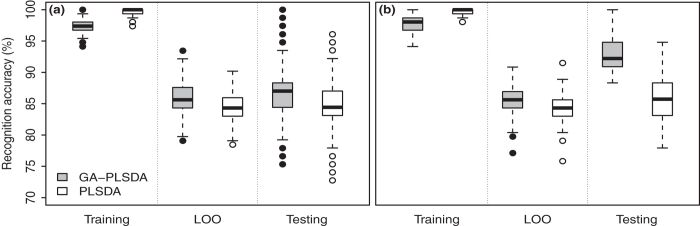
Comparison of correctness in calibration, cross-validation and prediction for PLSDA and GA-PLSDA with two different schemes.

When we re-sampled our training set, we verified that the PLSDA approach had consistently greater accuracy during calibration, but the GA-PLSDA approach produced higher accuracy during internal and external validation (Fig. 5). The subset of variables selected by the GA during our initial analysis performed well even with different training datasets (Fig. 5a). The median (with 95% CI) LOO validation and external validation classification accuracies for this GA-PLSDA were 85.6% (85.5%-85.8%) and 87.0% (86.8%-87.2%) respectively, a slight but consistent improvement over the corresponding accuracies of 84.3% (84.2%-84.5%) and 84.4% (84.2%-84.6%) obtained by PLSDA without variable selection. This indicates the features selected by the GA from the initial training data captured the key features from other possible training datasets. Recreating the GA variable selection process for each training dataset gave a virtually identical LOO validation accuracy of 85.6% (85.2%-86.0%), but external validation accuracy increased to 92.2% (91.6%-92.8%) (Fig. 5b). From this we conclude that the GA-PLSDA model building process is relatively robust to variance in the exact composition of the training and test datasets, although using variables appropriate to the training data results in substantial improvement. This provides additional evidence that GA variable selection identifies features of real and meaningful importance to the system being studied.

In summary, we have demonstrated the capacity of NIR spectral data coupled with PLSDA to recognize soils according to its category with a high degree of accuracy. The use of GA variable selection improved recognition accuracy through PLSDA, and we demonstrated that variable selection was only minimally impacted by sampling effects. With the easily-obtained NIR spectrum of a soil, the already-established model is ready to be used as a reference tool to quickly distinguish between different soils types and place each soil type into its correct category. While our approach achieved very respectable recognition accuracy, there are several aspects of this method that could be improved. Although the method of maximum distance we used to classify individual samples is straightforward and easily implemented, other methods such as centroids or Mahalanobis distances between soil types might lead to improvements in classification accuracy. Alternative methods, such as nonlinear iterative partial least squares followed by linear discriminant analysis (NIPALS-LDA), may also perform better^17^. Data mining should always be explored to improve the accuracy and predictive models for soil type recognition, especially if more spectral data are available for the continued development across larger spatial scales. Finally, the GA method is based on stochastic processes and finding a globally optimal solution with a large number of variables and samples may require unreasonable computational resources. Although there may be only a local optimum, the set of variables selected by the GA greatly reduced the dimensionality of our input data, permitting us to focus our interpretation on a relatively narrow segment of the NIR spectrum.

## Methods

### Field description and soil properties

**Figure 6:**
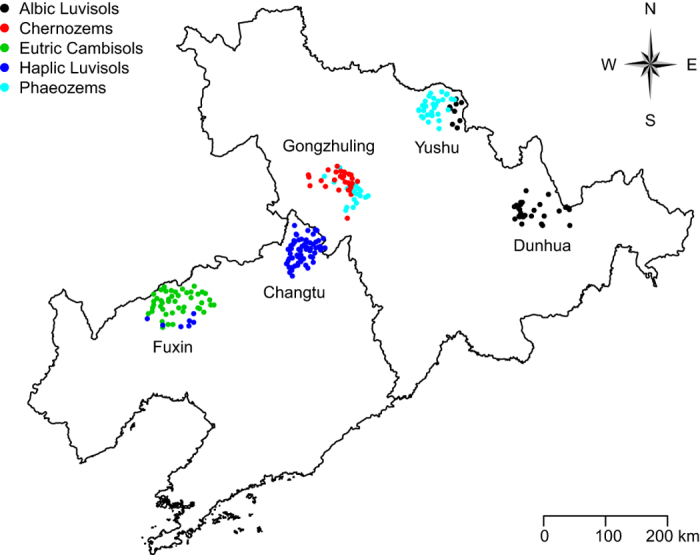
Distribution of soil sampling locations across five regions in northeastern China. Points denote individual sample sites. Note: the files (R format) that were used for map creation were free of charge and downloaded from the website http://www.gadm.org/country; the map was generated using “maptools” package in R.

Soil samples (*n* = 230) were collected from farmland at 0-10 cm depth in northeastern China in fall 2011. Sampling was conducted over five counties including Dunhua, Changtu, Gongzhuling, Fuxin, and Yushu, located at 41°N to 42°N latitude and 121°E to 128°E longitude, with the exact sampling locations shown in the map (Fig. 6). The climate is relatively cool and humid, with mean annual precipitation of 350–700 mm and mean annual air temperature of 3-9 °C for the five counties. Dominant soil types of this area are: Albic Luvisols, Haplic Luvisols, Chernozems, Eutric Cambisols and Phaeozems according to the FAO soil classification system^38^. All studied soils were manually pre-identified and classified to provide as supervised information for model establishment. The soil samples were air dried, sieved through a 0.25 mm sieve and visible identifiable crop residues were manually removed for further analysis. We analyzed soil organic carbon using dry combustion method by an element analyzer (vario MACRO cube, Elementar Analysensysteme GMbH, Hanau, Germany) and soil texture using a pipette method^39^.

### NIR spectra measurements

NIR spectrum absorbance bands were from first overtone and combination bands of the fundamental vibrations in the mid-infrared region^40^. Near infrared diffuse reflectance spectra were obtained from all soil samples (the average from three separate spectra for each sample) using a Thermo Nicolet 6700 spectrometer (Thermo Electron Scientific Instruments Corp., Madison, WI, USA). The background spectrum was eliminated automatically. The NIR spectra were recorded in the range of 7500 to 4000 cm^–1^ with 64 scans and 4 cm^–1^ resolution.

### Model development

Model development was carried out in the R statistical environment (Version 3.1.1)^41^. PLSDA model development and cross-validation were conducted using the “pls” package^42^, while the GA procedure was implemented following the scheme proposed by Wehrens (2011)^43^. In order to establish a valid and robust model, we randomly divided the soil sample data set into two parts: a training set for model development and cross-validation and a testing set for model external validation. For each soil type, samples were allocated between the training and testing sets along a 2:1 ratio, as described in the Supplementary. Since inherent features of all NIR spectra have no differences in magnitude, it was unnecessary to pretreat data prior to modeling.

PLSDA is a supervised classification method based on PLS regression^18^. In this study, the response matrix was conducted as a binary matrix having 5 columns with value 1 indicating group membership and 0 for non-membership. In order to predict values for multivariate response from a (potentially large) matrix of predictors; we used partial least squares regression (PLS2 algorithm), which calibrates all values in the response matrix simultaneously^44^, to develop the PLSDA model. We used a simple maximum distance rule to identify the class membership of each sample, which in this study meant a sample was assigned to the soil type that has the largest predicted value from the PLS2 regression model. We assessed the quality of the PLSDA models incorporating from 1 to 30 latent variables by using internal validation (leave-one-out (LOO) cross-validation) and external validation. For both methods, we used percentage classification accuracy as the metric of model performance. Variable importance in projection (VIP) scores were used to estimate the importance of each variable in the projection used in a PLS model^17^.

Genetic algorithms (GA) have several basic steps: 1) coding of the variable; 2) initiation of population; 3) evolution of the response; 4) reproduction; 5) mutation and 6) repeat the process until a stopping criterion is reached. Generally, the stopping criterion includes a maximum number of generations, a maximum target outcome value for the fitness or a set number of generations^45^. In this study, a simple GA procedure was used to select the minimum features important to PLSDA from the full set of NIR features. A very clear description of this process is given in section 2.4 of Ramadan *et al.*^30^. Briefly, we constructed a “population” consisting of sets of NIR features to include. Each subset was used in a PLSDA with a set number of latent variables, and assigned a fitness based on the following function:


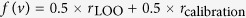


where *v* is the set of features included, *r*_calibration_ is the classification accuracy obtained during PLSDA calibration and *r*_LOO_ is the classification accuracy during LOO cross-validation. The variable sets with the highest fitness would be recombined with other fit sets to construct the next generation of the population. The resulting “offspring” would undergo “mutation” via random addition or removal of individual features to prevent the population from becoming trapped at a local optimum. The process continued until either a predetermined fitness value was reached or a maximum number of generations had elapsed. Each subset contained at least one more feature than the number of latent variables to be included in the subsequent PLSDA, with a maximum of 75 features. We used a population size of 50, a maximum of 20 generations, and a mutation probability of 0.05 per feature per generation.

### Model comparison

We evaluated the robustness of variable selection by GA to sampling biases by replicating the model fitting process 1000 times using re-sampled training datasets. For our test case (Scheme 1), we used a single set of GA-selected features with all training datasets, while in baseline case (Scheme 2), we used the GA to select a new set of features for each training dataset. Each of these cases was compared to PLSDA models using all features. All models fit during this processes used 26 latent variables.

## Additional Information

**How to cite this article**: Xie, H. *et al.* Soil type recognition as improved by genetic algorithm-based variable selection using near infrared spectroscopy and partial least squares discriminant analysis. *Sci. Rep.* **5**, 10930; doi: 10.1038/srep10930 (2015).

## Supplementary Material

Supplementary Information

Supplementary Dataset 1

Supplementary Dataset 2

## Figures and Tables

**Table 1:** The recognition rates of soil types using PLSDA

	**Albic Luvisols**	**Haplic Luvisols**	**Chernozems**	**Eutric Cambisols**	**Phaeozems**	**Classification accuracy (%)**
**Training**						
Albic Luvisols	24	0	0	0	0	100
Haplic Luvisols	0	48	0	0	0	100
Chernozems	0	0	19	0	0	100
Eutric Cambisols	0	0	0	30	0	100
Phaeozems	0	0	0	0	32	100
**Mean**						**100**
**LOO**						
Albic Luvisols	21	0	0	0	3	87.5
Haplic Luvisols	0	48	0	0	0	100
Chernozems	1	3	12	0	3	63.16
Eutric Cambisols	0	1	0	28	1	93.33
Phaeozems	0	2	3	0	27	84.38
**Mean**						**88.89**
**Testing**						
Albic Luvisols	12	0	0	0	0	100
Haplic Luvisols	1	15	0	6	2	62.50
Chernozems	0	0	9	0	1	90.00
Eutric Cambisols	0	2	0	13	0	86.67
Phaeozems	1	0	0	0	15	93.75
**Mean**						**83.12**

**Table 2:** The recognition rates of soil types using GA-PLSDA

	**Albic Luvisols**	**Haplic Luvisols**	**Chernozems**	**Eutric Cambisols**	**Phaeozems**	**Classification accuracy (%)**
**Training**						
Albic Luvisols	23	0	0	0	1	95.83
Haplic Luvisols	0	48	0	0	0	100
Chernozems	0	1	18	0	0	94.74
Eutric Cambisols	0	0	0	30	0	100
Phaeozems	0	1	0	0	31	96.88
**Mean**						**98.04**
**LOO**						
Albic Luvisols	21	2	0	0	1	87.50
Haplic Luvisols	0	47	0	0	1	97.92
Chernozems	1	2	15	0	1	78.95
Eutric Cambisols	0	1	0	29	0	96.67
Phaeozems	0	1	2	0	29	90.63
**Mean**						**92.16**
**Testing**						
Albic Luvisols	12	0	0	0	0	100
Haplic Luvisols	1	20	0	3	0	83.33
Chernozems	0	0	10	0	0	100
Eutric Cambisols	0	0	0	15	0	100
Phaeozems	0	0	1	0	15	93.75
**Mean**						**93.15**
