# Supplementary material for: Soil type recognition as improved by genetic algorithm-based variable selection using near infrared spectroscopy and partial least squares discriminant analysis
Source: Sci Rep. 2015 Jun 18;5:10930. doi: 10.1038/srep10930 (PMC4650675; doi:10.1038/srep10930)
Supplement: Supplementary Information [file srep10930-s1.pdf]

Soil type recognition as improved by genetic algorithm-based variable selection using near infrared spectroscopy and partial least squares discriminant analysis

Hongtu Xie[1,2], Jinsong Zhao[3], Qiubing Wang[4], Yueyu Sui[5], Jingkuan Wang[6], Xueming Yang[7], Xudong Zhang[1,7], Chao Liang[1,8,*]

[1]State Key Laboratory of Forest and Soil Ecology, Institute of Applied Ecology, Chinese Academy of Sciences, Shenyang, 110164, China

[2]Key Laboratory of Pollution Ecology and Environmental Engineering, Institute of Applied Ecology, Chinese Academy of Sciences, Shenyang, 110164, China

[3]College of Resources and Environment, Huazhong Agricultural University, Wuhan 430070, China

[4]College of Land & Environment, Shenyang Agricultural University, Shenyang 110866, China

[5]Northeast Institute of Geography and Agroecology, Chinese Academy of Sciences, Haerbin, 150000, China

[6]Greenhouse and Processing Crops Research Centre, Agriculture & Agri-Food Canada, Harrow, Ontario, N0R 1G0, Canada

[7]National Field Research Station of Shenyang Agroecosystems, Shenyang 110016, China

[8]Great Lakes Bioenergy Research Center, University of Wisconsin, Madison, 53706, USA

∗ Corresponding author. E-mail: cliang823@gmail.com;

Supplementary information

Table 1. Soil dataset used in this study

Site	Soil type	Samples	Training dataset	Test dataset
Dunhua	Albic Luvisols	36	24	12
Changtu	Haplic Luvisols	72	48	24
Gongzhuling	Chernozems	29	19	10
Fuxin	Eutric Cambisols	45	30	15
Yushu	Phaeozems	48	32	16
	Total	230	153	77

The essential function in R for PLSDA, and GA-based variable selection applied to soil type identification using NIR

```
##############
### PLS-DA ####
##############

class2ind <- function(cls) {
  numeric.class <- as.numeric(cls)
  M <- nlevels(cls)
  N <- length(cls)
  ind <- matrix(0, nrow = N, ncol = M)
  for (i in 1:N) {
    j <- numeric.class[i]
    ind[i, j] <- 1
  }
  colnames(ind) <- as.character(levels(cls))
  return(ind)
}

max.dist = function(x){
  nr = nrow(x)
  tmp = vector("numeric", nr)
  for(j in 1:nr){
    tmp[j] = (which(x[j, ] == max(x[j, ]))[1])
  }
  return(tmp)
}

# The main PLS-DA function
plsda <- function(X.train, cls.train, X.test, cls.test, ncomp, loo = TRUE, scale = FALSE, ...) {
```

```
  require(pls)
  if(!is.matrix(X.train)) X.train <- as.matrix(X.train)
  if(scale) {
    X.train <- scale(X.train)
    X.mean <- attr(X.train, "scaled:center")
    X.sd <- attr(X.train, "scaled:scale")
  }
  Y.train <- class2ind(cls.train)
  if(loo) {
    fm <- plsr(Y.train ~ X.train, ncomp, validation="LOO", ...)
  }
  else {
    fm <- plsr(Y.train ~ X.train, ncomp, ...)
  }

  train.pred <- fm$fitted.values
  cls.train.pred <- matrix(levels(cls.train)[apply(train.pred, 3, max.dist)], ncol = ncomp)
  colnames(cls.train.pred) <- paste("comp", 1:ncomp, sep = " ")
  tab.train <- tapply(cls.train.pred, col(cls.train.pred, as.factor = TRUE), function(j) table(factor(j, levels = levels(cls.train)), cls.train))
  ratio.train <- sapply(tab.train, function(tab) sum(diag(tab))/sum(tab))

  if (loo) {
    loo.pred <- fm$validation$pred
    cls.loo.pred <- matrix(levels(cls.train)[apply(loo.pred, 3, max.dist)], ncol = ncomp)
    colnames(cls.loo.pred) <- paste("comp", 1:ncomp, sep = " ")
    tab.loo <- tapply(cls.loo.pred, col(cls.loo.pred, as.factor = TRUE), function(j) table(factor(j, levels = levels(cls.train)), cls.train))
    ratio.loo <- sapply(tab.loo, function(tab) sum(diag(tab))/sum(tab))
  }
  else {
    cls.loo.pred <- NULL
    tab.loo <- NULL
    ratio.loo <- NULL
  }

  if(!missing(X.test)) {
    if(!is.matrix(X.test)) X.test <- as.matrix(X.test)
    if(scale) X.test <- scale(X.test, X.mean, X.sd)
    test.pred <- predict(fm, X.test)
    cls.test.pred <- matrix(levels(cls.train)[apply(test.pred, 3, max.dist)], ncol = ncomp)
    colnames(cls.test.pred) <- paste("comp", 1:ncomp, sep = " ")
    if(!missing(cls.test)) {
      tab.test <- tapply(cls.test.pred, col(cls.test.pred, as.factor = TRUE), function(j) table(factor(j, levels = levels(cls.train)), cls.test))
      ratio.test <- sapply(tab.test, function(tab) sum(diag(tab))/sum(tab))
    }
  }
  else {
    cls.test.pred <- NULL
```

```
    tab.test <- NULL
    ratio.test <- NULL
  }

  return(list(fm = fm, class.train.pred = cls.train.pred, class.test.pred = cls.test.pred, class.loo.pred =
cls.loo.pred, table.train = tab.train, table.test = tab.test, table.loo = tab.loo, ratio.train = ratio.train,
ratio.test = ratio.test, ratio.loo = ratio.loo))
}

train.selection <- function(x, ratio = 2/3) {
  tr.n <- floor(table(x) * ratio)
  tr.sel <- list()
  for (i in levels(x)) {
    tr.sel[[i]] <- sample(which(x == i), tr.n[i], replace = FALSE)
  }
  unlist(tr.sel, use.names = FALSE)
}

# Modified based on http://mevik.net/work/software/VIP.R #
VIP <- function(object, idx) {
  SS <- c(object$Yloadings[idx,])^2 * colSums(object$scores^2)
  Wnorm2 <- colSums(object$loading.weights^2)
  SSW <- sweep(object$loading.weights^2, 2, SS / Wnorm2, "*")
  sqrt(nrow(SSW) * apply(SSW, 1, cumsum) / cumsum(SS))
}

##########################
#####Genetic algorithms #####
##########################

# Following GA functions were extracted from package `ChemometricsWithR'
# with slight modification.
GA.init.pop <- function(popsize, nvar, kmin, kmax) {
  lapply(1:popsize,
      function(ii, x, min, max) {
        if (min == max) {
          sample(x, min)
        } else {
          sample(x, sample(min:max, 1))
        }
      },
      nvar, kmin, kmax)
}

GA.select <- function(pop, number, qlts, min.qlt = .4, qlt.exp = 1) {
  n <- length(pop)
  qlts <- qlts - min(qlts)
  threshold <- quantile(qlts, min.qlt)

  weights <- rep(0, n)
```

```
  if (any(OK <- qlts > threshold))
    weights[OK] <- qlts[OK] ^ qlt.exp
  else
    weights <- NULL

  sample(n, number, replace = TRUE, prob = weights)
}

GA.XO <- function(subset1, subset2) {
  n1 <- length(subset1)
  n2 <- length(subset2)
  if (n1 == n2) {
    length.out <- n1
  } else {
    length.out <- sample(n1:n2, 1)
  }
  sample(unique(c(subset1, subset2)), length.out)
}

GA.mut <- function(subset, nvar, kmin, kmax, mut.prob = .01) {
  if (runif(1) < mut.prob) { # swap variable in or out
    new <- sample((1:nvar)[-subset], 1) ###<<<--- why 1?
    length.out <- sample(-1:1 + length(subset), 1)
    if (length.out < kmin) length.out <- kmin
    if (length.out > kmax) length.out <- kmax
    sample(c(new, subset), length.out)
  } else {              # do nothing
    subset
  }
}

# The main GA function
GA.fun <- function(X, C, X.test, C.test, eval.fun, kmin, kmax, popsize = 50, niter = 200, mut.prob = .05,
min.qlt = .8, qlt.exp = .25, bar = TRUE, ...) {
  nvar <- ncol(X) # preparations: the first generation
  pop <- GA.init.pop(popsize, nvar, kmin, kmax)
  pop.q <- sapply(pop, function(subset) eval.fun(X, C, subset, X.test, C.test, ...))
  best.ini <- max.min(pop.q, pop)
  best.q <- best.ini$best.q
  best <- best.ini$best
  if (bar) pb <- txtProgressBar(1, niter, style=3)
  for (i in 1:niter) { # Go!
    new.pop <- lapply(1:popsize,
                 function(j) {
                   GA.mut(GA.XO(pop[[GA.select(pop, 1, pop.q, min.qlt = min.qlt, qlt.exp = qlt.exp)]],
                                pop[[GA.select(pop, 1, pop.q, min.qlt = min.qlt, qlt.exp = qlt.exp)]]),
                          nvar, kmin, kmax, mut.prob = mut.prob)
                 }) # do crossover, and later perhaps mutation
    pop <- new.pop
    pop.q <- sapply(pop, function(subset) eval.fun(X, C, subset, X.test, C.test, ...))
```

```
        best.i <- max.min(pop.q, pop)
        if (best.i$best.q == best.q) {
            if (length(best.i$best) < length(best)) {
                best <- best.i$best
            }
        }
        if (best.i$best.q > best.q) {
            best.q <- best.i$best.q
            best <- best.i$best
        }
        if (bar) setTxtProgressBar(pb,i)
    }
    if (bar) close(pb)
    list(best = best, best.q = best.q, n.iter = i)
}

plsda.fun <- function(x, class, subset, x.test, class.test, ...) {
    plsda.model <- plsda(x[,subset], class, x.test[,subset], class.test, ...)
    l <- plsda.model$fm$ncomp
    ratio.train <- plsda.model$ratio.train[l]
    ratio.loo <- plsda.model$ratio.loo[l]
    ratio.test <- plsda.model$ratio.test[l]
    0.5 * ratio.loo + 0.5 * ratio.train
}

max.min <- function(x,y) {
    ind <- which(x == max(x))
    len <- sapply(y, length)
    len.ind <- len[ind]
    len.min <- which.min(len.ind)
    res.ind <- ind[len.min]
    best.q <- x[res.ind]
    best <- y[[res.ind]]
    return(list(best.q = best.q, best = best))
}

# The user-interface function
GA.result <- function(ncomp, popsize=50, niter=20, N = 100) {
    best <- list()
    best.q <- c()
    ratio <- matrix(NA, nrow = N, ncol = 3)
    pb <- txtProgressBar(1, N, style=3)
    for (i in 1:N) {
        tmp <- GA.fun(tr.data, tr.class, test.data, test.class, eval.fun = plsda.fun, ncomp = ncomp, kmin =
ncomp+1, kmax = 75, popsize = popsize, niter = niter, bar = FALSE)
        fm <- plsda(tr.data[,tmp$best], tr.class, test.data[,tmp$best], test.class, ncomp = ncomp)
        ratio[i, ] <- c(fm$ratio.train[ncomp], fm$ratio.loo[ncomp], fm$ratio.test[ncomp])
        best[[i]] <- tmp$best
        best.q[i] <- tmp$best.q
        setTxtProgressBar(pb,i)
```

```
    }
    close(pb)
    colnames(ratio) <- c("train", "loo", "test")
    list(best = best, best.q = best.q, ratio = ratio)
}

###################
### Permutation #####
###################
plsda.scheme.1 <- function(X, cls, idx.c, N=1000, ncomp) {
  ratio <- matrix(NA, nrow = N, ncol = 6)
  idx <- list()
  pb <- txtProgressBar(1, N, style=3)
  for (i in 1:N) {
    idx.r <- train.selection(cls)
    idx[[i]] <- idx.r
    tmp.ga <- plsda(X[idx.r, idx.c], cls[idx.r], X[-idx.r, idx.c], cls[-idx.r], ncomp = ncomp)
    tmp.ori <- plsda(X[idx.r,], cls[idx.r], X[-idx.r,], cls[-idx.r], ncomp = ncomp)
    ratio[i,c(1,3,5)] <- c(tmp.ga$ratio.train[ncomp], tmp.ga$ratio.loo[ncomp], tmp.ga$ratio.test[ncomp])
    ratio[i,c(2,4,6)] <- c(tmp.ori$ratio.train[ncomp], tmp.ori$ratio.loo[ncomp], tmp.ori$ratio.test[ncomp])
    setTxtProgressBar(pb,i)
  }
  close(pb)
  list(ratio = ratio, idx = idx)
}

plsda.scheme.2 <- function(X, cls, ncomp, N= 1000, popsize = 50, niter = 20) {
  ratio <- matrix(NA, nrow = N, ncol = 6)
  idx <- list()
  ga <- list()
  pb <- txtProgressBar(1, N, style=3)
  for (i in 1:N) {
    idx.r <- train.selection(cls)
    idx[[i]] <- idx.r
    tmp <- GA.fun(X[idx.r,], cls[idx.r], X[-idx.r,], cls[-idx.r], eval.fun = plsda.fun, ncomp = ncomp, kmin = ncomp+1, kmax = 75, popsize = popsize, niter = niter, bar = FALSE)
    ga[[i]] <- tmp
    idx.c <- tmp$best
    tmp.ga <- plsda(X[idx.r, idx.c], cls[idx.r], X[-idx.r, idx.c], cls[-idx.r], ncomp = ncomp)
    tmp.ori <- plsda(X[idx.r,], cls[idx.r], X[-idx.r,], cls[-idx.r], ncomp = ncomp)
    ratio[i,c(1,3,5)] <- c(tmp.ga$ratio.train[ncomp], tmp.ga$ratio.loo[ncomp], tmp.ga$ratio.test[ncomp])
    ratio[i,c(2,4,6)] <- c(tmp.ori$ratio.train[ncomp], tmp.ori$ratio.loo[ncomp], tmp.ori$ratio.test[ncomp])
    setTxtProgressBar(pb,i)
  }
  close(pb)
  list(ratio = ratio, idx = idx, ga = ga)
```

```
}
?
```
